# Anti-apoptotic genes and non-coding RNAs are potential outcome predictors for ulcerative colitis

**DOI:** 10.1007/s10142-023-01099-9

**Published:** 2023-05-18

**Authors:** Wei Meng, Kay-Martin Johnsen, Christopher G. Fenton, Jon Florholmen, Ruth H. Paulssen

**Affiliations:** 1 Clinical Bioinformatics Research Group, Department of Clinical Medicine, Faculty of Health Sciences, UiT- The Arctic University of Norway, Tromsø, Norway; 2 Gastroenterology and Nutrition Research Group, Faculty of Health Sciences, UiT- The Arctic University of Norway, Tromsø, Norway; 3 Department of Medical Gastroenterology, University Hospital of North Norway, Tromsø, Norway; 4 Genomics Support Centre Tromsø, UiT- The Arctic University of Norway, Department of Clinical Medicine, Faculty of Health Sciences, UiT- The Arctic University of Norway, Sykehusveien 44, N-9037 Tromsø, Norway

**Keywords:** Ulcerative colitis, Remission, Cox analysis, Biomarkers

## Abstract

Due to the lack of clinical, immunologic, genetic, and laboratory markers to predict remission in ulcerative colitis (UC) without relapse, there is no clear recommendation regarding withdrawal of therapy. Therefore, this study was to investigate if transcriptional analysis together with Cox survival analysis might be able to reveal molecular markers that are specific for remission duration and outcome. Mucosal biopsies from patients in remission with active treatment-naïve UC and healthy control subjects underwent whole-transcriptome RNA-seq. Principal component analysis (PCA) and Cox proportional hazards regression analysis were applied to the remission data concerning duration and status of patients. A randomly chosen remission sample set was used for validation of the applied methods and results. The analyses distinguished two different UC remission patient groups with respect to remission duration and outcome (relapse). Both groups showed that altered states of UC with quiescent microscopic disease activity are still present. The patient group with the longest remission duration and no relapse revealed specific and increased expression of antiapoptotic factors belonging to the MTRNR2-like gene family and non-coding RNAs. In summary, the expression of anti-apoptotic factors and non-coding RNAs may contribute to personalized medicine approaches in UC by improving patient stratification for different treatment regimens.

**Supplementary Information:**

The online version contains supplementary material available at 10.1007/s10142-023-01099-9.

## Introduction

Ulcerative colitis (UC) is a chronic inflammatory disorder which requires long-term treatment in order to achieve remission (Ungaro et al. 2017). The inflammation status of UC patients is usually determined by endoscopic, histologic, and laboratory parameters (Peyrin-Biroulet et al. 2014; Rogler et al. 2013). Different guidelines for medical and surgical treatment of UC are available (Dassopoulos et al. 2015; Magro et al. 2017). In general, a step-up approach is recommended with the goal of obtaining clinical remission (Danese et al. 2014). Biological therapy is recommended for patients with moderate to severe disease refractory or patients dependent on steroid treatment. Side effects of both types of medication are common. The current management programs for UC aim for induction and maintenance of clinical remission to prevent treatment-induced and disease-related complications.

Today, different scoring systems for UC activity are in use to evaluate endoscopic disease activity and activity status, but none of the scoring systems have had all criteria fully determined (Travis et al. 2011; Rutter et al. 2004). There is no validated current definition of remission, and therefore still no consensus on how to define clinical remission (Magro et al. 2017). The guidelines from the European Colitis and Crohn’s organization (ECCO) for remission suggest the absence of visible mucosal lesions (Mayo endoscopic grade 0) in remission (Magro et al. 2013), whereas others allow Mayo ≤ 1 including endoscopic grade 1 in remission (Lamb et al. 2019; Rutgeerts et al. 2005; Schroeder et al. 1987). However, it is generally accepted that healed mucosa with the absence of mucosal lesions is a treatment goal. “Histological” healed mucosa is not included in clinical remission, but there is an increasing focus of including histological criteria in healed mucosa (Peyrin-Biroulet et al. 2014). It is well known that even in the absence of gastrointestinal symptoms as well as normal endoscopic and clinical findings, patients may have persisting microscopic inflammatory activity even in the absence of gastrointestinal symptoms (Korelitz 2010; Magro et al. 2018; DeRoche et al. 2014). This activity can result in progressive accumulation of bowel damage, such as fibrosis, dysmotility, and increased risk of colorectal neoplasm (Gupta et al. 2007).

It is self-evident that there is a need for standardization of both assessment and validation as well as prognostic values. There is still a need to characterize the complex pathogenic and healing mechanisms in UC. Due to the lack of clinical, immunologic, genetic, and laboratory markers to predict remission without relapse, there is no clear recommendation regarding withdrawal of therapy. Therefore, the current study aims to identify molecular signatures in a UC remission cohort obtained by whole-transcriptome RNA-Seq with the intent to provide a better understanding of the molecular mechanisms responsible for remission duration and disease outcome. Altogether, this knowledge might lead to novel personalized therapeutic approaches that will help patients to stay in remission.

## Materials and methods

### Patient material

A standardized sampling method was used to collect mucosal biopsies (*n *= 56) from patients in remission (RR; *n *= 26). For comparison purposes, normal patient biopsies (NN; *n *= 16) and biopsies from patients with active UC (UC; *n *= 14) were adapted from an earlier study (Fenton et al. 2021). The level of inflammation in UC patients was diagnosed based upon established clinical endoscopic and histological criteria as defined by the European Colitis and Crohn’s Organization (ECCO) guidelines (Magro et al. 2017). A total Geboes score was determined for the remission samples (Geboes et al. 2000). TNF mRNA levels in biopsies were estimated by qPCR (Olsen et al. 2007). TNF-α values of <7000 copies/ug RNA were considered non-inflamed tissues. Faecal calprotectin was measured with the Calprest ELISA kit (Eurospital). All patient characteristics are depicted in Table 1. All methods were performed in accordance with the Declaration of Helsinki. The study participants signed informed and written consent forms. Approvals were granted by the Regional Committee of Medical Ethics of Northern Norway, Ref no: 14/2004, 1349/2012 and 29895/2020. The samples were taken from an established biobank approved by the Norwegian Board of Health (952/2006).

**Table 1:** Characteristics of patients

Characteristics	Control^§^ (*n* = 16)	Remission (*n* = 26)	Treatment-naïve active UC^§^ (*n* = 14)
Gender (male/female)	11/5	15/11	9/5
Age (years) mean ± SD	52.5 ± 16.9	48.4 ± 13.4	40.7 ± 13.9
Endo score mean ± SD	0	0	1.79 ±0.43
Geboes score (total) ± SD	n.d.	0.36 ± 1.38	6.35 ± 2.93
TNF-α copies/μg RNA ± SD	3663 ± 1973	5060 ± 3047*	15907 ± 9623
Calprotectin (mg/kg) mean ± SD	n.d.	23.8 ± 35.7^€^	587.5 ± 483.8^¥^
Extension of disease^£^	_	2/7/8/9	2/9/3
Duration of remission (years) ± SD	_	4.38 ± 4.28	_
Medication^#^	_	26/0/7/2	_

*TNF-α copies/μg RNA in 18 patients. ^£^Proctitis/rectosigmoid/left-sided colitis/pancolitis. ^#^5-ASA/steroids/immunosuppressives/biologics. ^€^Average calprotectin levels in 16 patients. ^¥^Average calprotectin levels in 11 patients. ^**§**^Data adapted for comparison from Fenton et al. (Fenton et al. 2021)

### RNA isolation

Total RNA was isolated using the Allprep DNA/RNA Mini Kit from Qiagen (catalogue number 80204) and the QIAcube instrument (Qiagen), according to the manufacturer’s protocol. Quantity and purity of the RNA were assessed by using the NanoDrop ND-1000 spectrophotometer (Thermo Fisher Scientific, Wilmington, DE). The Experion Automated Electrophoresis System (Bio-Rad, Hercules, CA) and the RNA StdSens Analysis Kit (Bio-Rad, catalogue # 700–7103) were used to evaluate RNA integrity. The RNA samples were kept at −70 °C until further use. All RNA samples used for analyses showed an RNA integrity number (RIN) value of between 8.0 and 10.0.

### Library preparation and next-generation sequencing

Whole transcriptome libraries of UC remission samples were prepared with the TruSeq Stranded Total RNA LT Sample Prep Kit from Illumina (Catalogue number RS-122–2203). The amount of input material was 1 μg of total RNA. The Bioanalyzer 2100 (Agilent Technologies, Santa Clara, CA) and the Agilent DNA 1000 kit (Catalogue number 5067-1504) were used to assess RNA library quality, according to the instruction manual. The libraries were normalized to 10 nM and subsequently paired- end sequenced with the NextSeq 550 instrument (Illumina) according to the manufacturer’s instructions. The average number of uniquely mapped reads per sequencing run was 85 million reads per sample.

### Data analysis

**Fig. 1:**
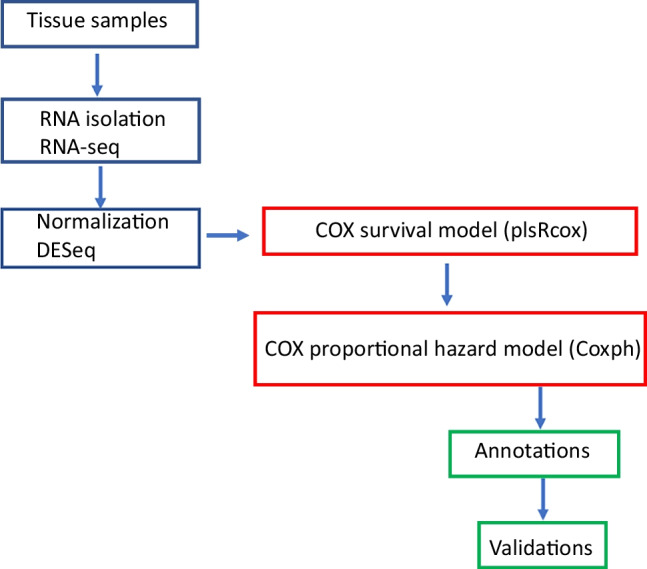
Study design. The flow chart depicts the entire workflow of the study

The entire design and workflow of the study is depicted in Fig. 1.

### Data quality assessment and initial principal component analysis (PCA)

Quality scoring and base calling were performed on the Illumina NextSeq 550 sequencing instrument. The output FastQ file was aligned with reference GENCODE Human Release 33 (Human Genome Assembly GRCh38.p13) (https://www.ncbi.nlm.nih.gov/grc/human/data) by STAR (Version 2.7.3a) with 2-pass mapping and gene counts parameters in STAR (Dobin et al. 2013). After alignment, the read quality was controlled by multiQC (Ewels et al. 2016). The gene counts were analysed and log-normalized by DESeq2 (Love et al. 2014); genes with an average log2 expression less than 4 were filtered out prior to normalization. Seven remission samples were randomly chosen for verification. Initial principal component analysis (PCA) was performed based on the top 15,000 variable genes after normalization.

Processed RNA-Seq data have been deposited in NCBI’s Gene Expression Omnibus (GEO, https://www.ncbi.nlm.nih.gov/geo/) and are accessible through GEO series accession numbers GSE128682 and GSE169360.

### Cox survival analysis of remission samples

After PCA, the remission patient group was investigated with Cox survival analysis in R using plsRcox (Bastien et al. 2015). Using remission patient information (Table 1) indicating state (relapse or not) and duration (time to relapse), a Cox model was created. The Cox model was applied on the normalized gene count matrix from remission patients. The initial Cox model was significant with a *p *< 0.01 in the likelihood ratio test and *p* = 0.03 in the Wald test for all normalized genes in the remission group, thus, suggesting that there is enough information in the gene matrix to explain patient risk. To further identify which genes influence risk for relapse, the R package survival (coxph) was then applied on each individual gene (Therneau 2021). The second analysis revealed 287 genes that significantly contribute to risk with a *p* value< 0.01. Those 287 genes were used for PCA analysis and visualization. Hazard beta-coefficients were calculated for the 23 selected genes.

### Annotations

Genes were manually annotated using GeneCards (https://www.genecards.org/). EnrichGO of the clusterProfiler R package (Yu et al. 2012) was applied to the protein-coding genes. Only biological process GO terms for comparisons of patient groups with *p*_adj_ < 0.05 were kept.

### Validations

Prior to analysis, seven remission samples were excluded from the remission patient cohort for validation. Gene counts from the four MTRNR2-like family genes (MTRNR2L6, MTRNR2L3, MTRNR2L12, and MTRNR2L8) from the validation samples were tested using the plsRcox package (Ginestet 2011). The statistics of all the Cox models are shown in Table S2.

### Data visualization

Heatmaps were generated by ComplexHeatmap (Gu et al. 2016). Among 287 genes, protein coding genes and non-protein coding genes were ranked by means of each gene in the remission samples divided by the sum of means of each gene in each group, respectively. The rows were clustered for better visualization.

## Results

### Transcriptomic analysis discriminates different states of UC

**Fig. 2:**
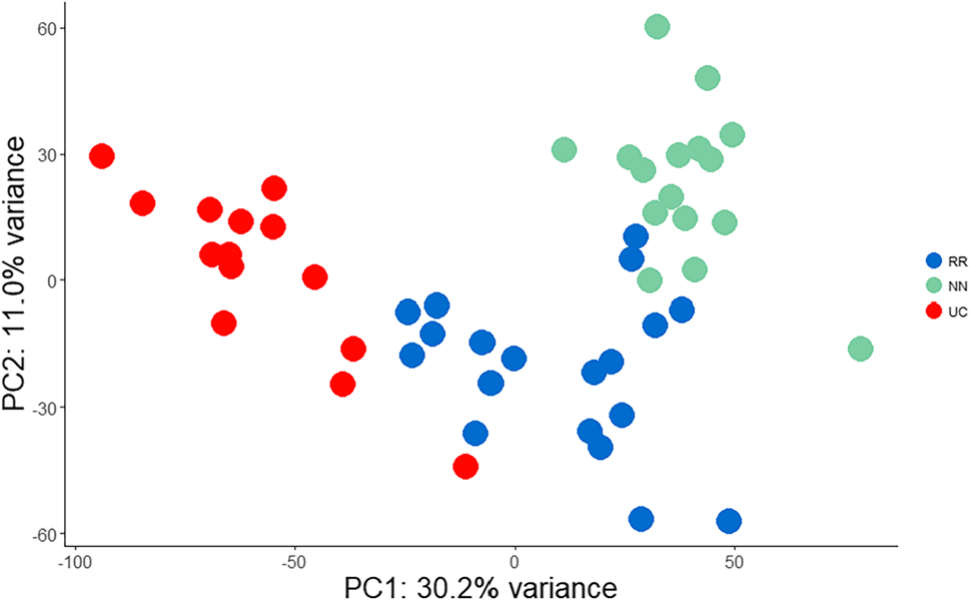
Principal component analysis (PCA) of remission, ulcerative colitis, and normal control patient samples. PCA of remission (RR), ulcerative colitis (UC), and normal control patient samples (NN) of the 15,000 most variable genes after normalization. Principal component (PC1) explained 30.4% of the total variance, and principal component 2 (PC2) explained 11% of the total variance

The whole transcriptome representing treatment-naïve active UC (UC; *n *= 14), UC in remission (RR; *n *= 19), and normal control samples (NN; *n *=16) was established by RNA-seq. Pre-processing of the sequencing data revealed a total of 18,783 expressed genes. The normalization of the expression of gene matrices for all groups showed no batch effects (Table S1). The initial principal component analysis (PCA) with 15,000 most variable genes resulted in a clear distinction between normal (NN), ulcerative colitis (UC), and remission (RR) samples, along the first principal component (PC1) with a 30.2% explained variance and an 11% explained variance along with the second component (PC2) (Fig. 2). Note, that prior to the initial PCA analysis, seven remission patient samples were randomly removed from the remission data set for validation of a Cox survival model (see below).

### Cox survival analysis discriminates genes related to remission duration and state

PCA alone did not result in a separation of remission samples, although they differed in terms of remission duration and time of relapse. Therefore, an attempt was made to distinguish remission samples by using different Cox models. Using the remission patient characteristics depicted in Table 1, a Cox survival model based on partial least squares was established using remission duration as survival time and relapse as an event. The model returned a likelihood test and Wald test *p* < 0.05 (Table S2), thus suggesting that there is enough information in the gene matrix to explain patient risk.

### Remission significant genes obtained by Cox analysis

**Fig. 3:**
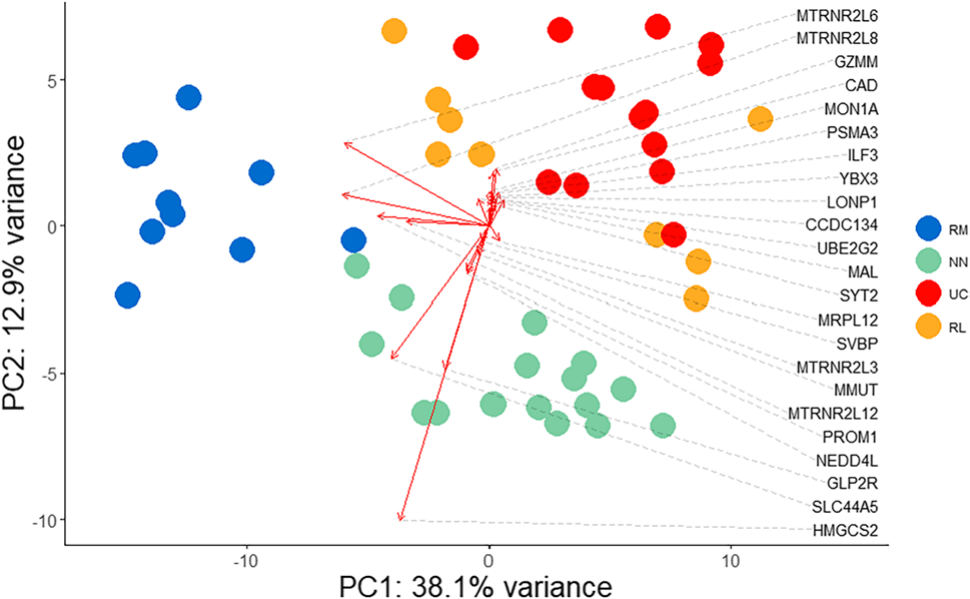
Principal component analysis of genes revealed by Cox analysis. Genes revealed from the Cox regression analysis (*n *= 287) were used for principal component analysis (PCA) including remission (RR), ulcerative colitis (UC), and normal control patient samples (NN). Principal component 1 (PC1) explained 38.1% of the total variance, and principal component 2 (PC2) explained 12.9% of the total variance. The biplot depicts 23 protein-coding genes of 188 protein-coding genes obtained by Cox analysis. The arrows indicate the genes as loading projectiles that differ the group from the direction. The length of each arrow represents the effect of genes on the components. To improve the visibility, the loadings were multiplied by 25. Each arrow is labelled with a gene name as indicated. An entire list of genes can be found in Table S3. Figure S1 depicts a biplot including all protein-coding genes

The second Cox analysis for each individual gene of the remission gene matrix returned 287 significant genes *p* <0.01 related to risk (Tables S2 and S3). Of the 287 genes, 188 represented protein-coding genes, 28 small RNAs, 25 non-coding RNAs, 31 pseudogenes, and 15 miscellaneous RNAs, which are all listed in Table S3. Significant genes (*n *= 287) obtained by the remission Cox analysis were visualized by PCA with co-normalized normal control samples (NN) and UC samples (UC) included. The result of the PCA shows that the significant genes from of the Cox model can clearly separate the remission samples into two groups with 38.1% and 12.9% of explained variances for principal component 1 (PC1) and principal component 2 (PC2) (Fig. 3). Both remission groups showed clear differences with respect to endoscopic, histological, and laboratory parameters and were then denominated accordingly RM (remission without relapse) and RL (remission with relapse) (Fig. 3 and Table S4). The PCA biplot shows both PC scores of samples (dots) and loadings of variables (vectors). The further away these vectors are from a PC origin, the more influence they have on that PC. Notably, the RM samples grouped closer to the normal control samples, whereas RL samples clustered and in part overlapped with UC samples (Fig. 3).

### Genes of the MTRNR2-like family separate remission duration

**Fig. 4:**
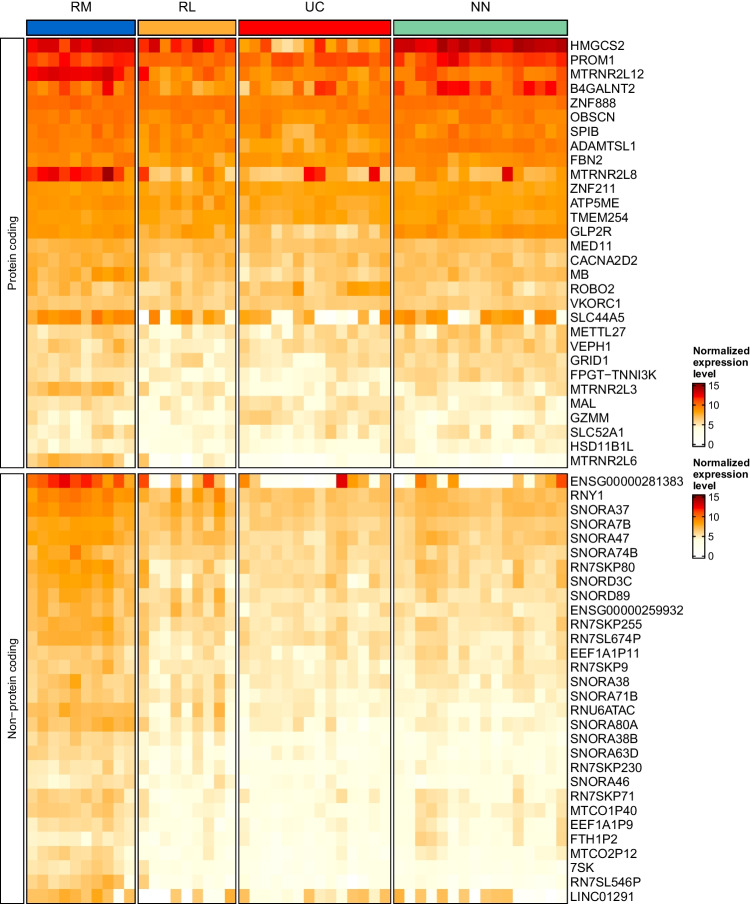
Heatmap of selected genes of relevance for remission status. Heatmaps were generated by ComplexHeatmap as described in the “Materials and methods” section. Thirty protein coding genes and non-protein coding genes were ranked by means of each gene in the remission samples divided by the sum of means of each gene in each group, respectively. Long-term remission samples (RM), short-term remission samples (RL), treatment-naïve ulcerative colitis samples (UC), and normal control samples (NN) are depicted and normalized expression levels of a genes are indicated

Two biplots were constructed on the PCA, one including all 188 protein-coding genes and one including 99 non-coding genes indicating the effect of each individual gene on the principal components (Figs. S1 and S2). For illustrative purposes, twenty-three relevant protein-coding genes with high influence were chosen for construction of a biplot (Fig. 3). In addition, the expressions of relevant 30 protein-coding genes and non-coding genes found for the different patient groups were visualized in a heatmap (Fig. 4).

Genes of the MTRNR2-like family influenced the remission samples separation. Solute carrier family 44 member 5 (SLC44A5), glucagon like peptide 2 receptor (GLP2R), prominin 1 (PROM1), NEDD4 like E3 ubiquitin protein ligase (NEDD4L), and methylmalonyl-CoA mutase (MMUT) are the main participants for components for separation towards the normal control group. Genes like interleukin enhancer binding factor 3 (ILF3), carbamoyl-phosphate synthetase 2, aspartate transcarbamylase, and dihydroorotase (CAD), Mal, T cell differentiation protein (MAL), and granzyme M (GZMM) are influencing the separation of UC samples and RL samples.

### Hazard values confirm separation and specificity of expressed genes

**Fig. 5:**
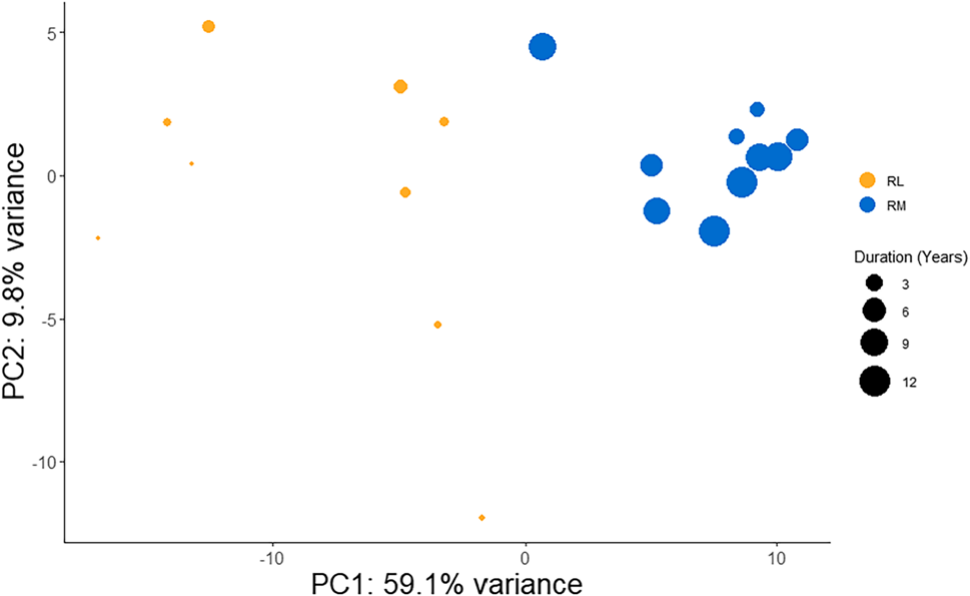
Separation of UC remission samples by PCA. Separation of remission samples by principal component analysis (PCA) using 287 genes obtained by Cox analysis. The samples separate into two groups dependent on remission duration, remission without relapsing (RM, blue), and remission with relapsing (RL, yellow). The size of the circles represents the duration of remission. Principal component 1 (PC1) explained 59.1% of the total variance, and principal component 2 (PC2) explained 9.8% of the total variance

**Fig. 6:**
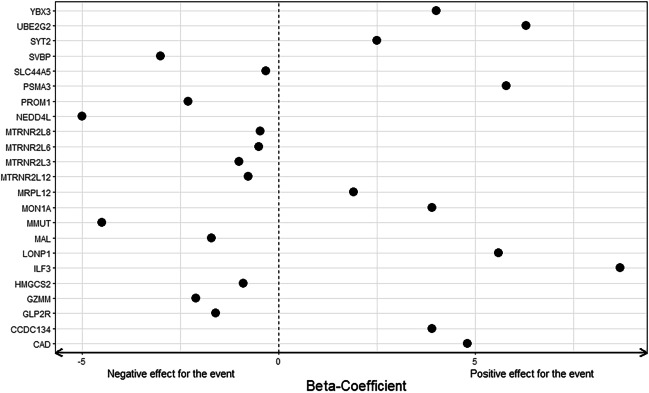
Beta-coefficients obtained by Cox proportional hazards regression analysis. Beta-coefficients indicate the contribution of each gene to the relative risk of relapse in the Cox survival analysis for 23 UC-relevant genes shown in Fig. 2. The figure shows the beta-coefficient value on the *X*-axis for each gene. Zero is marked as a dashed line. A negative value indicates a protective effect of a gene with which it is associated, and vice versa

To confirm this finding, a PCA using the 287 genes from the remission gene count matrix only was performed (Fig. 5). The result confirmed the separation and specificity of the expressed genes with 59.1% and 9.8% of explained variances for PC1 and PC2 in the remission matrix PCA. Beta-coefficients (hazard values) for selected 23 individual genes are shown in Fig. 6. Ten genes including ILF3, mitochondrial ATP-dependent protease Lon (LONP1), proteasome 20S subunit alpha 3 (PSMA3), and CAD were found to increase the chance of relapse which is reflected by negative coefficients. Thirteen genes including MTRNR2 like family, PROM1, and NEDD4L decrease the probability of relapse which is reflected by positive coefficients. A complete beta value list of all genes (*n *= 287) can be found in Table S5.

### Annotation reveals involvement of apoptotic and RNA processing pathways

**Fig. 7:**
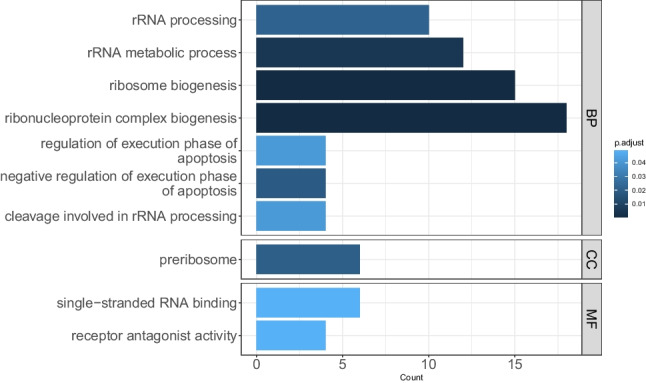
Functional annotations of genes revealed by Cox analysis. The protein-coding genes were annotated with gene ontology (GO). Enriched pathways and genes involved are indicated. The length of bars indicates the number of genes involved in the GO terms for biological process (BP), cellular component (CC), and molecular function (MF). A complete list of enriched GO pathways annotations can be found in Table S6

Among the 287 genes, 188 were protein-coding genes. GO enrichment of these 188 protein-coding genes is shown in Fig. 7. Significantly enriched gene sets revealed biological processes like negative regulation of the execution phase of apoptosis with genes of the MTRNR2-like family (MTRNR2L6, MTRNR2L8, MTRNR2L3, MTRNR2L12), ribosome biogenesis, rRNA processing, RNA splicing, signal transduction by p53 class mediator, and ribonucleoprotein complex biogenesis. The cellular component preribosome and molecular functions including single-stranded RNA binding and receptor antagonist activity were enriched. The full enrichment list is shown in Table S6.

### MTRNR2-like genes are predictors for risk of relapse

**Fig. 8:**
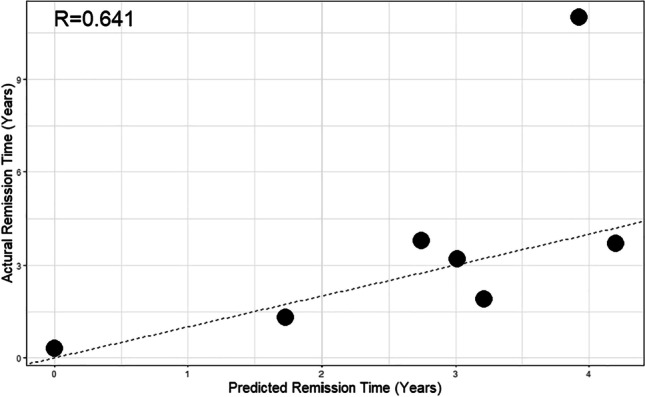
Validation of the Cox survival model. The validation of the Cox model was tested with seven UC remission samples. The correlation plot depicts the predicted remission time (years) on the *X*-axis and the actual remission time (years) on the *Y*-axis for a group of 7 randomly picked patient samples using a gene set including 4 MTRNR-like genes (MTRNR2L6, MTRNR2L3, MTRNR2L12, MTRNR2L8). The correlation between the two parameters is estimated at *R*= 0.641

Seven remission testing samples were used to validate the influence of the four MTRNR2-like genes (MTRNR2L6, MTRNR2L8, MTRNR2L3, and MTRNR2L12) using the Cox model. The correlation between predicted duration and actual duration *R*= 0.641 (Fig. 8). This indicates that the MTRNR2-like genes are good predictors for risk of relapse.

## Discussion

Today, the recommendations regarding the withdrawal of therapy during UC are not clear. Therefore, the current study aimed to identify molecular signatures in a UC remission patient cohort with focus on remission duration after treatment and disease outcome. The analysis of transcriptional expression data of UC remission samples obtained by RNA-Seq, Cox survival analysis, and downstream PCA analysis of genes obtained by Cox survival analysis clearly revealed a relationship between remission event (duration of remission) and remission states (relapse or no relapse). Initial PCA analysis (Fig. 2) on the normalized expression matrix of all patient samples confirmed similar distribution patterns for remission samples found for two independent UC remission cohort studies studying the differential expression of genes, showing a clear distinction between UC in remission, normal controls, and active UC samples (Fenton et al. 2021; Planell et al. 2013). PCA analysis of the genes obtained by Cox analysis could clearly separate remission samples into two groups representing UC remission, one with relapse (RL) and one without relapse (RM) (Figs. 3 and 5). The Cox analysis, using the remission gene matrix only, showed that the model was independent of the other sample groups, UC and NN (Fig. 5). Therefore, it is surprising that a clear relationship between selected genes and the UC and NN background samples could be observed (Fig. 3).

The obtained molecular signatures did show different inflammatory states in the remission groups (Fig. 3, Tables S3 and S4). A quiescent inflammation is still present in remission which is reflected by the expression of interleukin enhancer binding factor 3 (ILF3) which is involved in innate immune responses and myeloid dendritic cell maturation in IBD (Aitchison et al. 2021). The influence shown in the biplot (Figs. 3 and S1) on the first principal component and a high beta coefficient found for ILF3 confirms inflammatory signals in RL samples (Fig. 6 and Table S5). Likewise, increased expression of other inflammatory genes like CAD which is involved in the inhibition of NOD2 antibacterial function in intestinal epithelial cells (Richmond et al. 2012) and PSMA3 which is involved in the proteasome-mediated NF-dB activation in UC (Goetzke et al. 2021) was observed. Recently, a relationship between UC and atherosclerosis has been implicated (Weissman et al. 2020; Roifman et al. 2009). The reported higher risk of cardiovascular events in UC patients may be pertinent in inflammation-mediated atherosclerosis (Rungoe et al. 2013; Kristensen et al. 2013). The mitochondrial matrix protein LONP1 has been shown to be involved in atherosclerosis mitochondrial protein quality control (Hansen et al. 2008; Onat et al. 2019) and is a strong risk factor of relapse (Fig. 6). All the above-mentioned genes are shown to have an influence pointing towards inflammation and increased risk of relapse especially for patients in the RL group (Figs. 3, 6, and S1).

It is well-known that mitochondrial function in the intestinal epithelium plays a critical role in maintaining intestinal health (Urbauer et al. 2021). A recent paediatric UC patient cohort study revealed suppressed expression of mitochondrial genes in active UC (Haberman et al. 2019). The here observed increased expression of MTRNR2-like genes might improve the remission state (Fig. 3, Table S3, Fig. S1). Mitochondrial dysfunction and dysbiosis of gut microbiota have been shown to be associated with IBD (Jackson and Theiss 2020). Therefore, a recovery of the gut-microbiota environment and restoring of rectal mitochondrial energy functions can be implied for remission without relapse (RM) where commensal bacterial-induced mitochondrial signalling potentiates epithelial homeostasis. The specific expression of MTRNR2-like genes in RM might represent these genes as potential molecular markers for disease outcome (Figs. 3 and 4, and Table S3). The GO annotations confirmed enrichment of genes involved in the regulation of execution phase of apoptosis (Fig. 7).

It is interesting to note that MTRNR2 treatment may exert beneficial effects in UC by decreasing inflammatory reactions and apoptosis (Gultekin et al. 2017). The mitochondrial metabolism in the intestinal stem cell niche plays also a pivotal role in regulating intestinal epithelial cell homeostasis, including self- renewal and differentiation (Urbauer et al. 2021). The observed expression of stem cell marker prominin 1 (PROM1) (Karim et al. 2014) and NEDD4 like E3 ubiquitin protein ligase (NEDD4L) points to a maintenance of proliferation and differentiation of the colonic epithelium in RM (Kimura et al. 2011) (Figs. 3 and 4). NEDDL4 strongly contributes to a lower risk of relapse (Fig. 6). In addition, increased expression of the vitamin B12 dependent, mitochondrial MMUT (Park et al. 2021) in RM points to a lower B12 deficiency reported for UC patients thereby lowering the risk of relapse (Fig. 6) (Battat et al. 2014; Mortimore and Florin 2010).

Top genes with great influences towards normal control samples in the PCA are HMGSCS2, BAGALNTT2, and GLP2R (Figs. 3 and S1). HMGCS2 encodes a mitochondrial protein that belongs to the HMG-CoA synthase family and catalyses the first reaction of ketogenesis. Elevated expression of HGMCS2 has been reported recently for long-duration ulcerative colitis (Low et al. 2019). Here, HMGCS2 showed increased expression in both remission groups when compared to UC and contributes to a lower risk of relapse (Table S3 and Fig. 6). However, a high expression of HMGCS2 has been associated with the development of colorectal cancer (CRC) which is contrary to these findings (Chen et al. 2017). Increased expression of glycosyltransferase B4GALNT2 in RM points to a maintenance of the intestinal mucus barrier function (Table S3) (Bergstrom et al. 2017). The increased expression of GLP2R involved in the stimulation of intestinal growth, increase of crypt cell proliferation and decrease of enterocyte apoptosis by glucagon-like peptides, prevents intestinal hypoplasia (Drucker 2003).

Interestingly, nearly all the non-coding genes shown in the biplot demonstrate an influence towards RM and normal controls in the PCA (Fig. S2). The expression of 20 small nucleolar RNAs (snoRNAs) (Fig. 4, Table S3, and Fig. S2) may be involved in the mediation of cell–cell communication and improvement of cell survival in the face of stress and/or infection (Rimer et al. 2018), and long non-coding RNAs (lncRNAs) have been shown to have relevance for ulcerative colitis pathogenesis (Ghafouri-Fard et al. 2020; Yarani et al. 2018; Ray et al. 2022). Functions of non-coding RNAs in ribosomal RNA (rRNA) regulation have been recently reported where especially snoRNAs and long non-coding RNAs play important roles in pre-rRNA transcription, processing, and maturation (Li et al. 2013). These pathways are shown to be enriched in RM (Fig. 7).

However, the relevance of specific expression of non-coding RNAs for UC remission duration and outcome needs further evaluation. In this context, it is interesting to note that synergistic gene regulation by pseudogenes and non-coding RNAs has been considered a novel regulatory mechanism which might have a role in UC pathogenesis (Li et al. 2013; Milligan et al. 2015).

This study is not without limitations and is limited by a restricted number of patient samples. Yet, a separation in the PCA after Cox analysis was clearly derived (Figs. 3 and 5). Although several studies present gene expression data of UC patients in remission, separate patient samples with indicated time of relapse were not available for validation of the Cox model. Knowing that the sample number was low, the Cox survival model was then validated with 7 randomly chosen remission patient samples and could confirm the model (Fig. 8) using four MTRNR2-like genes. In addition, a patient cohort with the possibility to investigate the remission state in the same patients consecutively was not available at the time of this study. Nevertheless, the different remission groups do not resemble a normal control phenotype. Patients in the RM group that have been previously treated with anti-TNF therapy (infliximab) until endoscopic remission and subsequently been treated with 5-aminosalicylic acid (5-ASA) only did not experience relapse (Johnsen et al. 2017). Patients in the RL remission group remained in remission for up to 8 months with additional immunosuppressive treatment but had a relapse at some point during the treatment period.

## Conclusions

The data clearly demonstrate that remission is an altered state of UC with quiescent microscopic disease activity still present. This disease activity is independent of remission duration and outcome. Transcription expression analysis and Cox survival analysis revealed potential markers genes that could be useful to predict disease outcome. These markers include mitochondrial MTRNR2-like genes and non-coding RNAs. Especially, the expression of anti-apoptotic factors and snoRNAs may contribute to personalized medicine approaches in UC by improving patient stratification for different treatment regimens. The data presented might be of clinical utility in the future.

## Supplementary information

ESM 7 Figure S1: Biplots of 188 protein-coding genes. (PDF 505 kb)

ESM 8 Figure S2: Biplot of 99 non-protein coding genes. (PDF 407 kb)

ESM 1 Table S1: Normalization of the expression gene matrices of all patient groups. (PDF 31 kb)

ESM 2 Table S2: Statistics of the used Cox models. (PDF 229 kb)

ESM 3 Table S3: All 287 genes from the Cox model on gene expression level. (XLSX 42 kb)

ESM 4 Table S4: Patients characteristics of two separate remission patient groups (DOCX 15 kb)

ESM 5 Table S5: Beta values obtained for each gene. (XLSX 23 kb)

ESM 6 Table S6: GO enrichment of 287 genes with adjusted p value< 0.05. (XLSX 11 kb)

\

## Data Availability

The dataset generated and analysed during the current study is available at GEO accession number GSE169360: Go to https://www.ncbi.nlm.nih.gov/geo/query/acc.cgi?acc=GSE169360. Enter token ovgloyiyptofdud into the box. All other data generated or analysed during this study are included in this published article and supplementary files.
