# Supplementary material for: Anti-apoptotic genes and non-coding RNAs are potential outcome predictors for ulcerative colitis
Source: Funct Integr Genomics. 2023 May 18;23(2):165. doi: 10.1007/s10142-023-01099-9 (PMC10195737; doi:10.1007/s10142-023-01099-9)
Supplement: Supplementary file 1 — Figure S1: Biplots of 188 protein-coding genes. (PDF 505 kb) [file 10142_2023_1099_MOESM1_ESM.pdf]

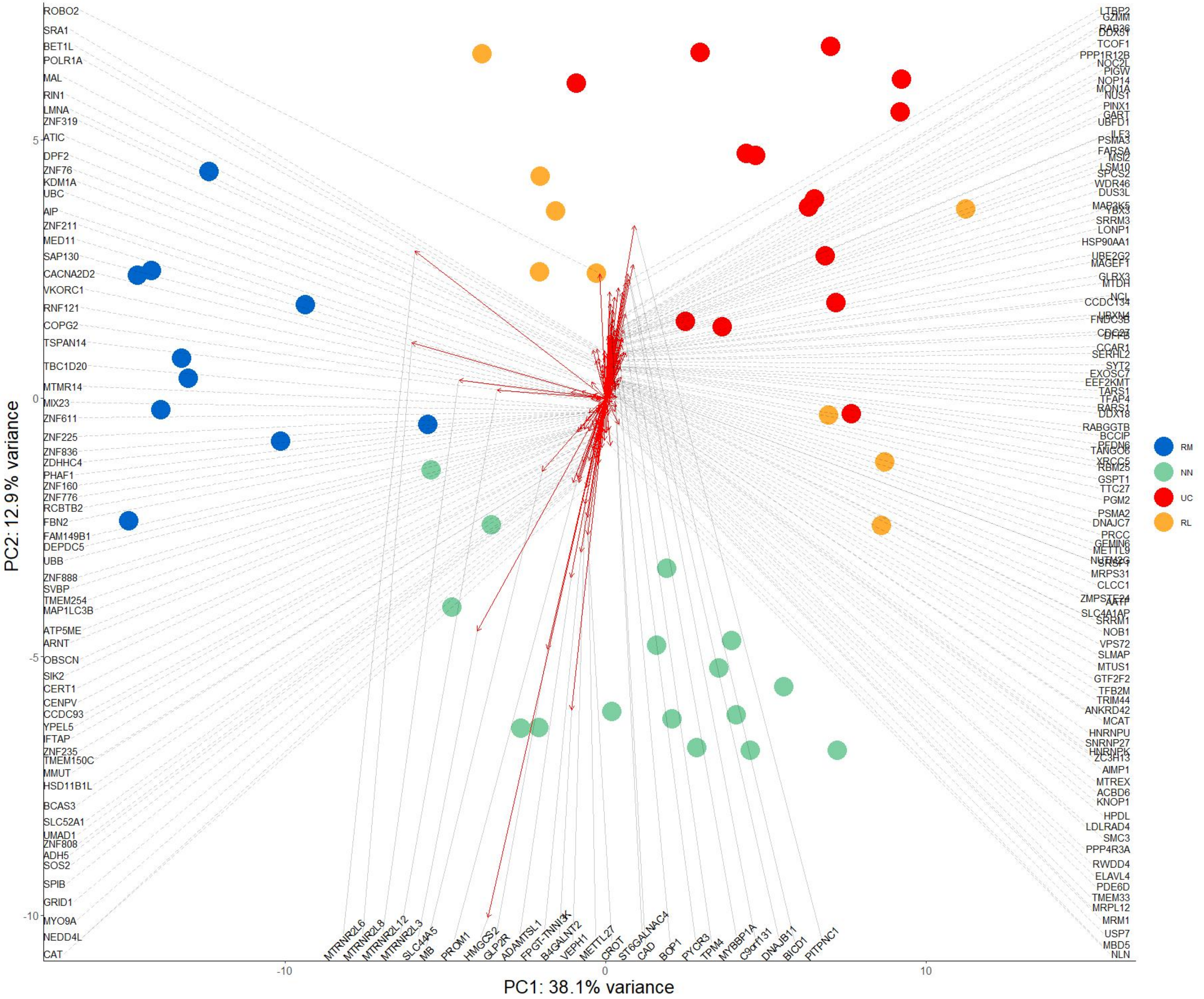

Figure S1. Biplot of long-term remission (RM, blue), short-term remission (RL, orange), ulcerative colitis (UC, red) and normal control patient samples (NN, green) of the 287 genes. Principal component (PC1) explained 38.1 % of the total variance, and principal component 2 (PC2) explained 12.9 % of the total variance. Red arrows represent 188 protein coding genes.
