# Supplementary material for: Anti-apoptotic genes and non-coding RNAs are potential outcome predictors for ulcerative colitis
Source: Funct Integr Genomics. 2023 May 18;23(2):165. doi: 10.1007/s10142-023-01099-9 (PMC10195737; doi:10.1007/s10142-023-01099-9)
Supplement: Supplementary file 3 — Table S1: Normalization of the expression gene matrices of all patient groups. (PDF 31 kb) [file 10142_2023_1099_MOESM3_ESM.pdf]

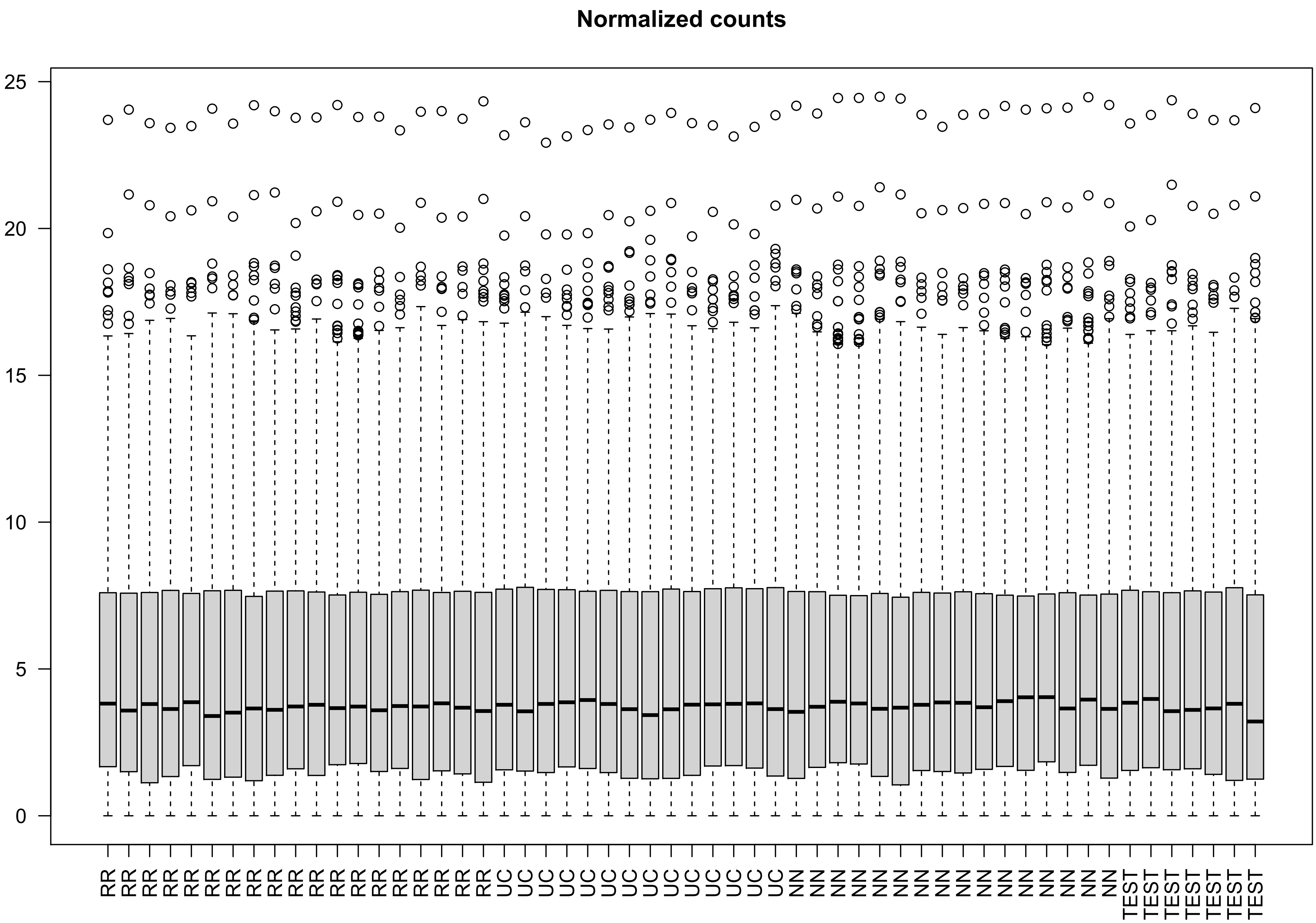

Normalization of the expression of gene matrices for all groups, ulcerative colitis (UC), remission (RR) and normal samples (NN)
