# Supplementary material for: Anti-apoptotic genes and non-coding RNAs are potential outcome predictors for ulcerative colitis
Source: Funct Integr Genomics. 2023 May 18;23(2):165. doi: 10.1007/s10142-023-01099-9 (PMC10195737; doi:10.1007/s10142-023-01099-9)
Supplement: Supplementary file 4 — Table S2: Statistics of the used Cox models. (PDF 229 kb) [file 10142_2023_1099_MOESM4_ESM.pdf]

Statistics of the **used** Cox models

- **The Cox model on 18783 genes:**

```
  n= 19, number of events= 9

         coef exp(coef) se(coef)      z Pr(>|z|)
dim.1 -0.10908   0.89666  0.04383 -2.489   0.0128 *
dim.2 -0.00158   0.99842  0.01216 -0.130   0.8966
---
Signif. codes:  0 '***' 0.001 '**' 0.01 '*' 0.05 '.' 0.1 ' ' 1

      exp(coef) exp(-coef) lower .95 upper .95
dim.1    0.8967      1.115    0.8228    0.9771
dim.2    0.9984      1.002    0.9749    1.0225

Concordance= 0.904  (se = 0.056 )
Likelihood ratio test= 22.18  on 2 df,   p=2e-05
Wald test            = 7.13  on 2 df,   p=0.03
Score (logrank) test = 16.18  on 2 df,   p=3e-04
```

- **Cox model on 289 genes:**

```
  n= 19, number of events= 9

        coef exp(coef) se(coef)      z Pr(>|z|)
dim.1 -0.3981    0.6716   0.1479 -2.691  0.00712 **
dim.2 -0.1435    0.8663   0.1159 -1.239  0.21545
---
```

Signif. codes: 0 ‘***’ 0.001 ‘**’ 0.01 ‘*’ 0.05 ‘.’ 0.1 ‘ ’ 1

	exp(coef)	exp(-coef)	lower .95	upper .95
dim.1	0.6716	1.489	0.5026	0.8975
dim.2	0.8663	1.154	0.6903	1.0872

Concordance= 0.952 (se = 0.027)

Likelihood ratio test= 31.07 on 2 df,   p=2e-07

Wald test   = 7.37 on 2 df,   p=0.03

Score (logrank) test = 26.52 on 2 df,   p=2e-06

- **Cox model on 4 genes (MTRNR2-like family):**

n= 19, number of events= 9

	coef	exp(coef)	se(coef)	z	Pr(>|z|)	
tt.1	0.7415	2.0990	0.2465	3.008	0.00263	**
tt.2	3.3678	29.0152	2.7578	1.221	0.22201	

Signif. codes: 0 ‘***’ 0.001 ‘**’ 0.01 ‘*’ 0.05 ‘.’ 0.1 ‘ ’ 1

	exp(coef)	exp(-coef)	lower .95	upper .95
tt.1	2.099	0.47641	1.2948	3.403
tt.2	29.015	0.03446	0.1304	6457.718

Concordance= 0.808 (se = 0.079)

Likelihood ratio test= 13.86 on 2 df,   p=0.001

Wald test   = 10.04 on 2 df,   p=0.007

Score (logrank) test = 14.62 on 2 df,   p=7e-04
