# Supplementary material for: Anti-apoptotic genes and non-coding RNAs are potential outcome predictors for ulcerative colitis
Source: Funct Integr Genomics. 2023 May 18;23(2):165. doi: 10.1007/s10142-023-01099-9 (PMC10195737; doi:10.1007/s10142-023-01099-9)
Supplement: Supplementary file 6 — Table S4: Patients characteristics of two separate remission patient groups (DOCX 15 kb) [file 10142_2023_1099_MOESM6_ESM.docx]

**Table S4:** Patient characteristics of UC remission groups RM and RL after Cox analysis.

| **Characteristics** | **Remission**  **no relapse (RM)**  **(n = 10)** | **Remission**  **relapse (RL)**  **(n = 9)** |
| --- | --- | --- |
| Gender (male/female) | 5/5 | 4/5 |
| Age (years) mean ± SD | 51.5 ± 10.7 | 48.8 ± 17.2 |
| Endo Score mean ± SD | 0 | 0.22 ± 0.44 |
| Geboes score (total) ± SD | 0.10 ± 0.31 | 0.44 ± 0.73 |
| TNF-α copies/µg RNA ± SD | 4862 ± 2697* | 5822 ± 4051 |
| Extension of disease^£^ | 2/5/1/2 | 0/2/5/2 |
| Duration of remission (years) | 7.6 ± 3.4 | 0.8 ± 0.5 |
| Medication^#^ | 10/0/0/0 | 9/0/5/1 |

*TNF-α copies/µg RNA in 9 patients

^£^proctitis/left-sided colitis/rectosigmoid/pancolitis

^#^5-ASA/steroids/immunosuppressives/biologics
